# The determining factor of a preferred orientation of GaN domains grown on *m*-plane sapphire substrates

**DOI:** 10.1038/srep16236

**Published:** 2015-11-09

**Authors:** Miyeon Jue, Cheol-Woon Kim, Seoung-Hun Kang, Hansub Yoon, Dongsoo Jang, Young-Kyun Kwon, Chinkyo Kim

**Affiliations:** 1 Department of Physics and Research Institute for Basic Sciences, Kyung Hee University, 26 Kyungheedae-ro, Dongdaemun-gu, Seoul 02447, Korea

## Abstract

Epitaxial lateral overgrowth in tandem with the first-principles calculation was employed to investigate the determining factor of a preferred orientation of GaN on SiO2-patterned *m*-plane sapphire substrates. We found that the (1
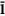
00)-orientation is favored over the (1
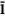
0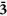)-orientation in the region with a small filling factor of SiO_2_, while the latter orientation becomes preferred in the region with a large filling factor. This result suggests that the effective concentration determines the preferred orientation of GaN: the (1
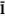
00)- and (1
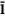
0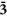)-orientations preferred at their low and high concentrations, respectively. Our computational study revealed that at a low coverage of Ga and N atoms, the local atomic arrangement resembles that on the (1
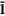
0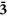) surface, although the (1
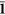
00) surface is more stable at their full coverage. Such a (1
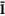
0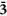)-like atomic configuration crosses over to the local structure resembling that on the (1
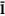
00) surface as the coverage increases. Based on results, we determined that high effective concentration of Ga and N sources expedites the growth of the (1
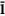
0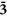)-orientation while keeping from transition to the (1
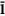
00)-orientation. At low effective concentration, on the other hand, there is a sufficient time for the added Ga and N sources to rearrange the initial (1
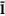
0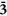)-like orientation to form the (1
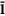
00)-orientation.

The performance of GaN-based optoelectronic devices have been gradually improved, but there are several issues still requiring further investigation to better understand this material, which would eventually help to enhance the performance of GaN-based devices. One of them is related to the growth of GaN films whose preferred orientation is not along the *c*-direction. When multiple quantum well structures are grown on *c*-plane sapphire substrates, the quantum confined Stark effect (QCSE), associated with the piezoelectric field and spontaneous polarization field, causes the separation of wave functions of electrons and holes in the quantum wells in such a way that the quantum efficiency decreases^1^,^2^. Unlike *c*-oriented GaN, nonpolar-oriented GaN such as *m*- or *a*-oriented GaN is supposed to have a significantly suppressed QCSE. While there are many reports on the growth and characterization of *a*-oriented GaN grown on *r*-plane sapphire substrates^3^,^4^, the papers on *m*-oriented GaN grown on *m*-plane sapphire substrates are very few partly because the preferred orientation of GaN grown on *m*-plane sapphire is not uniquely determined^5^,^6^,^7^.

Depending on the pretreatment conditions and the buffer layers used, GaN is known to have one of three possible orientations ((1
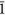
00), (1
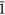
0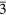), (11
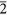
2)) as a preferred orientation when grown on *m*-plane sapphire substrates^8^,^9^,^10^. In addition to the pretreatment, the substrate temperature and growth parameters influence on the preferred orientation as well^7^,^11^. This dependence of the preferred orientation of GaN grown on *m*-plane sapphire on the experimental conditions draws prompt attention due to its technological importance in controlling the preferred orientation of GaN when grown on *m*-plane sapphire, but this intriguing dependence of preferred orientations on the experimental conditions is far from being scientifically understood. Therefore, it is of great importance to investigate the underlying mechanism of selection process of preferred orientation of GaN grown on *m*-plane sapphire by combining experimental and theoretical approaches. In this work, we employed both an epitaxial lateral overgrowth of GaN on SiO_2_-patterned *m*-plane sapphire substrates and *ab initio* density functional theory to investigate the determining factor of a preferred orientation of GaN domains when grown on *m*-plane sapphire substrates. We observed experimentally that the effective concentration of Ga and N species (*i.e*., the number of Ga and N species available for growth per unit opening area) may determine the preferred orientation, which was verified by our computational simulation, based on which we compared the formation energies of growing surfaces with different orientations.

## Results and Discussion

**Figure 1:**
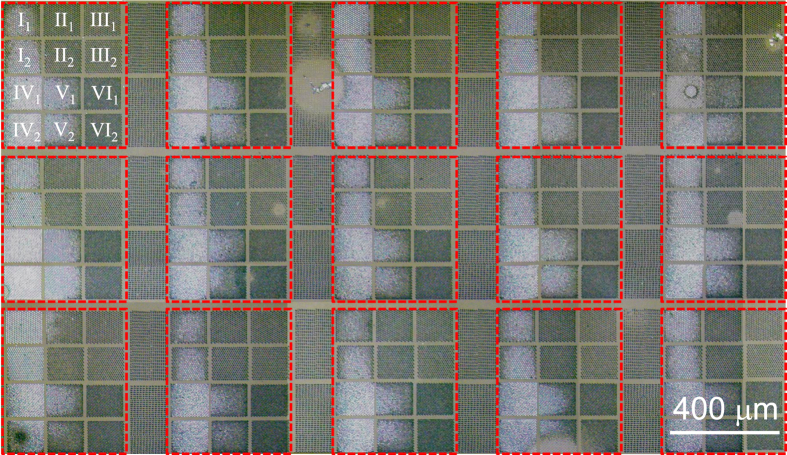
Optical microscopy image of laterally overgrown GaN on an SiO_2_-patterned *m*-plane sapphire substrate. The region enclosed by a red dashed rectangle is a unit cell of the pattern. Each unit cell consists of 12 divisions patterned with hexagonally arranged circular openings with different radii and separations. Each division labeled with a subscript 2 has a 90°-rotated pattern from the one in the division labeled with the same roman number and a subscript 1.

The unit cell of the mask pattern consists of 12 divisions as schematically shown in Fig. 1, which is a mosaic composite image obtained by joining together a series of optical microscopy images of epitaxially overgrown GaN. Each unit cell, enclosed by red dashed line segments, consists of 12 divisions patterned with hexagonally arranged circular openings. The radius of the circular openings is 2 *μ*m in division I and IV, 1.5 *μ*m in division II and V, and 1 *μ*m in division III and VI. The separation between the nearest circular openings is 8 *μ*m (in division I, II, and III) and 6 *μ*m (in division IV, V, and VI), respectively. The fractional ratio of an opening area (FROA) in hexagonally arranged circular openings is given by 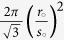, where *r*_°_ and *s*_°_ are a radius of a circular opening and a separation between circular openings, respectively. Thus, the FROA in each division is calculated to be 0.403(IV), 0.227(I and V), 0.128(II), 0.101(VI), and 0.0567(III). Note that the divisions I, IV, and V with higher FROA appear brighter than the other divisions with smaller FROA. It turned out that the brightness difference between divisions is closely related to site-dependent selection of preferred orientations of GaN domains, as discussed as follows.

**Figure 2:**
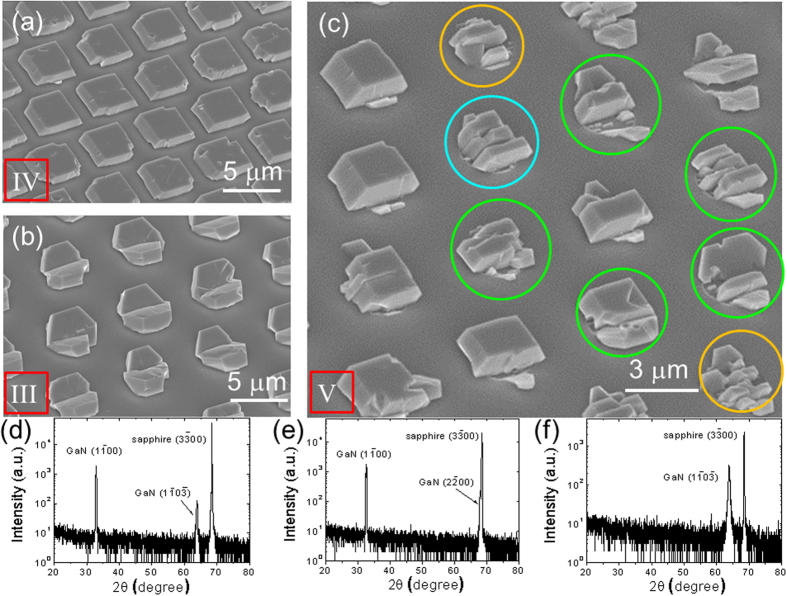
Secondary electron images of GaN domains shown in Fig. 1 with (a) (1
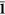
00), (b) (1
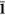
0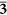), and (c) mixed orientations as preferred orientation. In (**c**), domains with both (1
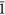
00) and (1
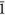
0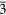)-orientation as preferred orientation are marked with green circles; the (1
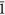
00)-oriented but not fully coalesced domains are marked with cyan circle; and orange circles represent those with unidentifiable preferred orientation as determined by visual inspection. XRD diffraction data for the samples (**d**) with mixed domains, (**e**) with domains like those in Fig. 2(**a**), and (**f**) with domains like those in Fig. 2(**b**). Thus, the determination of a preferred orientation by visual inspection and by X-ray diffraction is consistent with each other.

**Figure 3:**
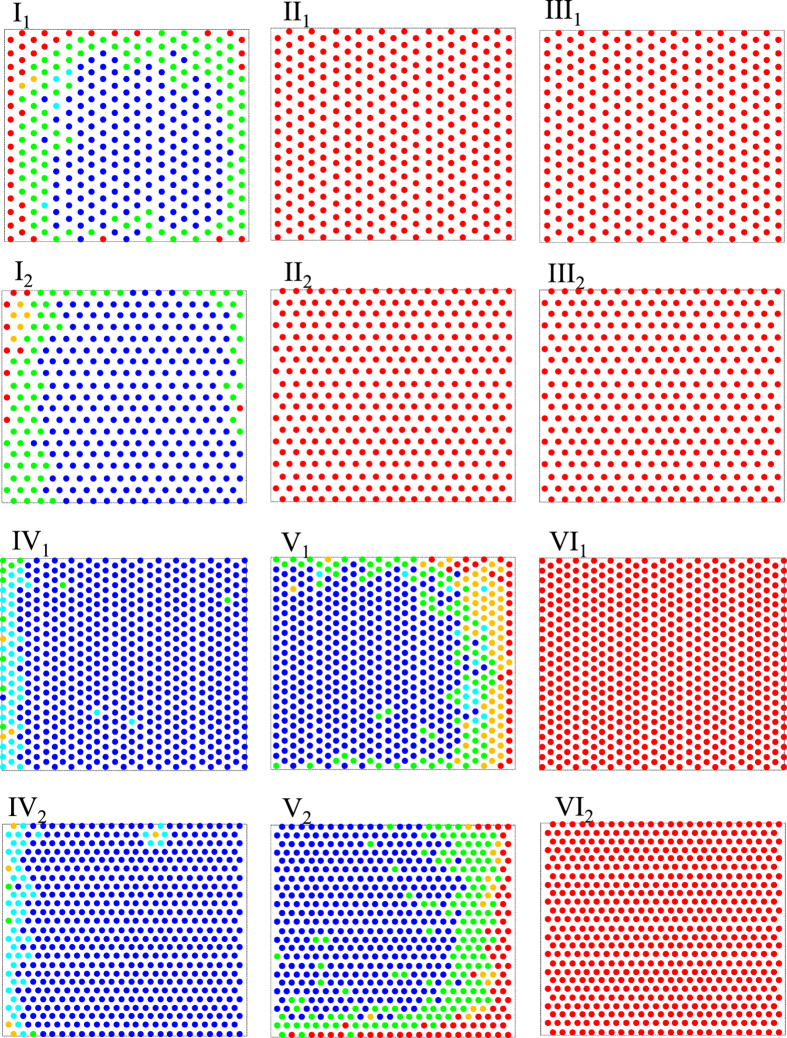
Color-coded preferred orientation distribution observed in one of the unit cells of the pattern by visual inspection. Blue and red represent domains with the (1
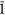
00)- and the (1
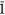
0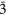)-orientation, respectively, while green is for the domains with both orientation direction as preferred orientation. Cyan is for the (1
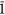
00)-oriented but not fully coalesced domains, and orange for the domains with underdeveloped facets of unidentifiable preferred orientation. Note that for clarity the colored circles in all divisions were drawn in the same size.

When GaN is grown on *m*-plane sapphire substrates, the crystallographic facets are typically well developed so that the preferred orientation can be readily recognized simply by visual inspection of a domain shape^12^,^13^. In Fig. 2, secondary electron images (SEIs) of GaN domains grown on an SiO_2_-patterned *m*-plane sapphire substrate illustrates domain shapes with various preferred orientations. From the visual inspection of a domain shape, a domain or domains in each circular hole could be categorized into five different groups: the (1
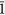
00)-oriented one (as illustrated in Fig. 2(a)), the (1
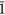
0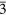)-oriented one (as illustrated in Fig. 2(b)), ones with both (1
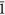
00)- and (1
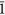
0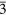)-orientation as a preferred orientation (as marked in Fig. 2(c) with green circles), the (1
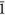
00)-oriented but not fully coalesced (as marked in Fig. 2(c) with cyan circle), one with visually unidentifiable preferred orientation (as marked in Fig. 2(c) with orange circles). Based on this categorization, the preferred orientation of a domain or domains in each circular hole was determined and its color-coded distribution was illustrated in Fig. 3.

We found that GaN domains in the divisions I, IV, and V with bright appearance mainly have the [1
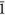
00] direction as a preferred orientation. On the other hand, the (1
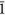
00)-oriented domains were not observed in the other divisions (II, III, and VI). Since the (1
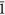
00)-oriented domain has the (1
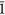
00) facet parallel with the surface of *m*-plane sapphire substrate, the domains in those divisions (I, IV, and V) appeared much brighter than those in the other divisions (II, III, and VI) because they transmitted much more light due to their flat top facet. Table 1 shows a summary of the preferred orientation distribution of GaN domains grown on an SiO_2_-patterned *m*-plane sapphire substrate with different FROA. It can be inferred from this table that the (1
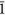
00) orientation was more favored than the (1
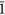
0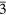) orientation in the region of a higher FROA.

**Figure 4:**
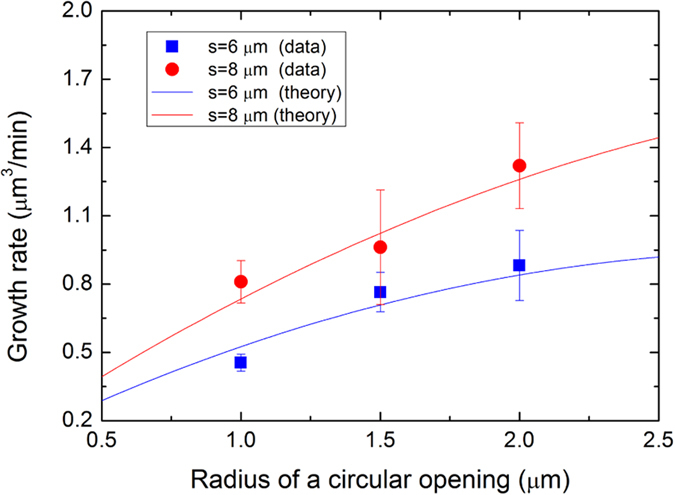
Experimentally obtained (symbols) and theoretically predicted (curves) growth rates of GaN domains as a function of a size of a circular opening with different opening separations.

In order to derive the relationship between FROA and the effective concentration of reacting species, we developed a simple model on the growth rate. Suppose that a reacting species is adsorbed at *r*, a distance from the center of a particular circular hole with its radius of *r*_°_. The probability of the adsorbate to contribute to the growth of the domain in that specific hole before it flies away can be assumed to be *r*_°_/*r* for *r* > *r*_°_ and 1 for *r* ≤ *r*_°_. Note that this assumption on the probability is justified in the following by the fact that the experimental growth rate is in good agreement with the theoretically predicted one based on this assumption. With a concentration of the reacting species per unit area to be *c*_°_, the total number of species contributing to the domain growth at each circular opening per unit time would be approximately given by *π r*_°_(*s*_°_ − *r*_°_)*c*_°_/*t*_°_ if the separation of circular opening and a growth time are given by *s*_°_ and *t*_°_. Thus, the effective concentration of reacting species per unit opening area would be proportional to (*s*_°_/*r*_°_ − 1)*c*_°_. The next step is to obtain the growth rate of GaN domains grown in different circular holes and to see if the experimental growth rates are in good agreement with the theoretically predicted one. The experimentally determined growth rates of GaN domains at openings with different FROA are plotted as symbols in Fig. 4, and the theoretically derived growth rates, which are proportional to *r*_°_(*s*_°_ − *r*_°_)*c*_°_/*t*_°_, are plotted as lines. The good agreement between the experimental growth rates and theoretically predicted ones corroborates that our model properly estimates the growth rates in this experimental configuration, which suggests that it is sufficiently reasonable to take (*s*_°_/*r*_°_ − 1)*c*_°_ as the effective concentration per unit opening area. Since FROA was found to be proportional to 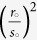 in the above, the effective concentration is inversely related to FROA. Thus, our experimental results can be interpreted in such a way that the (1
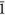
00) orientation was more favored as a preferred orientation than the (1
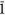
0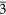) orientation at a low effective concentration of reacting species per unit area.

To scrutinize why the effective concentration of Ga and N plays a crucial role to determine the preferred orientation of GaN domains grown on *m*-plane sapphire substrate, we performed *ab initio* calculations based on DFT^14^,^15^,^16^. We first compared the stability at interfaces formed between one of two different GaN surfaces and the *m*-plane sapphire substrate in the following manner. In theoretical calculations, one cannot create a semi-infinite surface structure with one surface side, but can only construct a two-sided surface structure, unless some special techniques, such as Green function approach, are used. To deal with this issue, we constructed two superlattices composed of a GaN slab with either orientation and an m-plane sapphire slab. These slabs consist of four and six layers, respectively. We evaluated the work of separation associated with the interface energy by separating two slabs in each superlattice. The in-plane lattice constants (*a*, *b*) were selected to be (9.80, 12.99) and (19.61, 12.99) in a unit of Å for the (1
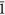
00) and (1
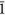
0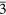) orientations, respectively. The lattice mismatches of the respective GaN films to the sapphire substrate were 8.3% and 2.4%. The thickness of the GaN film was 12.3 Å (11.3 Å) for the (1
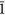
00) ((1
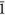
0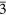)) orientation. While the GaN film is being separated from the substrate, we calculated the energy as a function of distance between the GaN film and the substrate. The works of separation were calculated to be 0.20 eV/Å^2^ and 0.18 eV/Å^2^ for the (1
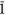
00) and (1
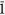
0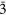) orientation, respectively. Their difference corresponds to ~4.3 eV when considered the size of the unit cell for the (1
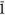
0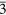) orientation, implying that the interface between (1
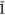
00)-oriented GaN film and *m*-plane sapphire substrate may be energetically much more stable than that of (1
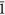
0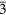)-oriented one and the substrate. However, such a thermodynamic argument does not provide any plausible explanation for the experimental observation of the less-stable (1
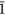
0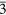)-oriented GaN surface.

**Figure 5:**
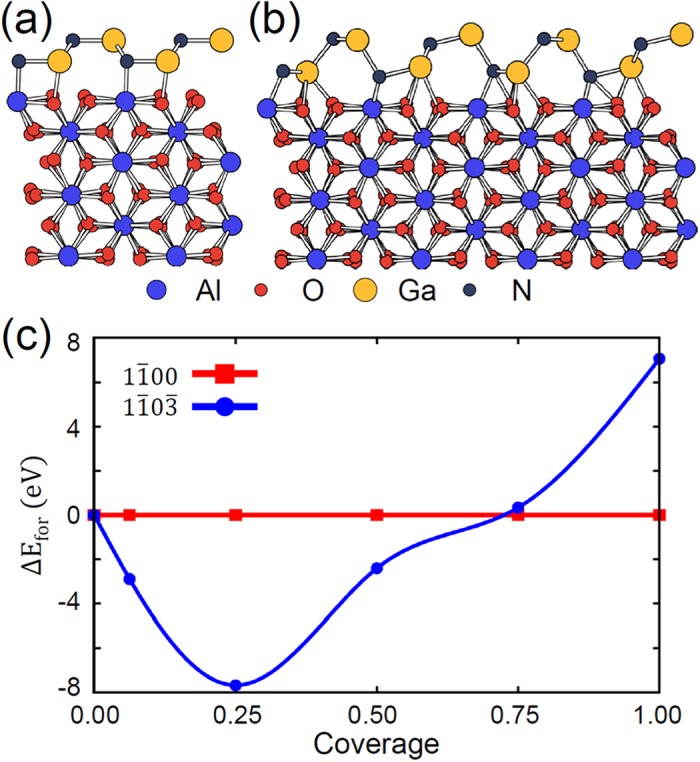
The model structure of GaN monolayers (full coverage) with (a) the (1
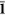
00)- and (b) the (1
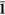
0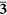)-orientation placed on *m*-plane of sapphire substrate modeled with 6 layers, the two bottom layers of which were fixed to mimic its bulk counterpart. (**c**) The formation energy of the (1
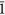
0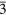)-oriented GaN film relative to that of the (1
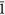
00)-oriented one, which was set to zero, as a function of GaN coverage.

To find a clue on this observation, we studied an early growth stage by exploring the structural stability of GaN adsorbed on *m*-plane sapphire substrate as a function of GaN coverage Θ while maintaining the local configuration of a particular surface orientation, either (1
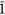
00) or (1
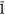
0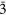). Figure 5(a, b) show the equilibrium structures of (1
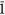
00)- and (1
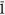
0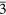)-oriented GaN single layers (Θ = 1) on *m*-plane sapphire substrate, respectively. We evaluated their formation energy as a function of Θ, and the surface energy for Θ > 1 as similarly done in an earlier study^17^. Figure 5(c) shows their relative formation energy, Δ*E*_f*or*_, with respect to the formation energy of the (1
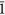
00)-oriented GaN case for each coverage value. Most intriguing feature in this graph is the presence of an energy crossover near the GaN coverage of Θ = 3/4. At lower coverage (Θ < 3/4), the (1
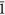
0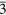) orientation case has lower formation energy indicating structurally more stable than the (1
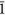
00) orientation, while at Θ > 3/4 the (1
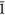
00) orientation exhibits lower formation energy, and thus becomes more stable than the other orientation. Based on our calculations, we can interpret our experimental observation as follows. At an early growth stage (Θ ≤ 3/4), Ga and N sources may tend to form patterns similar to the (1
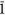
0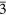)-oriented surface on the *m*-plane sapphire substrate. Around Θ = 3/4, the GaN growth would be split into two growth paths depending on the effective concentration of Ga and N species. The one path follows the thermodynamics at low effective concentration, since there would be sufficient time for adsorbed Ga and N atoms to be rearranged. Thus, the surface would experience a transition of the preferred orientation from the (1
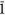
0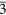) to the (1
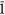
00) near Θ = 3/4. At a high effective concentration of Ga and N species, on the other hand, the surface growth continues in the (1
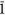
0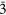) orientation even at Θ > 3/4, because more Ga and N atoms would be supplied prior to the transition to the (1
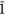
00) orientation, and thus maintain the (1
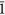
0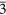) orientation. Nevertheless, the surface structure of the (1
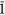
0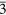) orientation, which is less stable than that of the (1
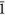
00) orientation, would form *m*- and *c*-facets at the growing surface to reduce the surface energy^18^, rather than keeping the flat surface with the (1
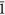
0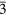) orientation.

In conclusion, we investigated on site-dependent selection of preferred orientation of GaN domains on SiO_2_-patterned *m*-plane sapphire substrates. Our investigation revealed that the effective concentration of reacting species (Ga and N) played an important role in determining a preferred orientation of GaN domains grown on *m*-plane sapphire substrates. We observed experimentally that the preferred orientation of GaN depended strongly on the effective concentration of reacting species. Our computational simulation disclosed that the preferred orientation of GaN grown on *m*-plane sapphire changes from (1
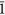
0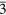) to (1
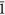
00) due to the crossover of the relative formation energy at a very low coverage of Ga and N species, is consistent with the experimental observation.

## Methods

GaN domains were laterally overgrown by utilizing hydride vapor phase epitaxy at 1000 °C on SiO_2_-patterned *m*-plane sapphire substrates with hexagonally arranged circular openings with various diameters (4, 3, and 2 *μ*m) and separations (8 and 6 *μ*m). The thickness of SiO_2_ pattern was 100nm. The flow rates of NH_3_ and HCl were 600 standard cubic centimeters per minute (sccm) and 4sccm, respectively. Nitrogen was used as a carrier gas. A total flow rate of N_2_ was 16.1 standard liter per minute (slm).

For *ab initio* calculations based on density functional theory (DFT), we adopted the basis consisting of pseudo-atomic orbitals (PAOs) generated by the split-valence scheme for a double-*ζ* polarized basis set, as implemented in the SIESTA code^19^,^20^. The Perdew-Burke-Ernzerhof (PBE) form^21^,^22^ was employed for the exchange-correlation functional in the generalized gradient approximation (GGA). The norm-conserving pseudopotentials of Troullier and Martins^23^ are used with the valence electrons of 3*d*^10^4*s*^2^4*p*^1^ for gallium, 2*s*^2^2*p*^3^ for nitrogen, 3*s*^2^3*p*^1^ for aluminum, and 2*s*^2^2*p*^4^ for oxygen. The charge density has been determined self-consistently on a real space mesh with a very high cutoff energy of 300 Ry, sufficient for total energy convergence to within 1 meV/atom. The energy shift due to the spatial confinement of the PAO basis functions^22^,^24^ was limited to less than 0.01 Ry.

Before constructing the model structures for our computational simulation, we reproduced the bulk structures of GaN (wurtzite) and sapphire (*α*-Al_2_O_3_), both of which are hexagonal with their respective lattice constants are *a*_GaN_ = 3.28 Å and *c*_GaN_ = 5.31 Å, and *a*_sap_ = 4.76 Å and *c*_sap_ = 12.99 Å in good agreement with previous experimental and theoretical results^25^,^26^,^27^. Then, we used supercells composed of GaN films and *m*-plane sapphire substrate, the latter of which was represented by six layers in a rectangular in-plane supercell with two lattice constants (*a*, *b*). We sampled the small quasi-2D Brillouin zones associated with the large supercells by 2 × 4 × 1 and 4 × 4 × 1 *k*-point meshes^28^ for (1
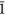
00) and (1
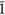
0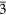) orientations, respectively. The equilibrium structures were obtained by performing geometry relaxation based on conjugate gradient method until the Hellmann-Feynman force acting on every atom became smaller than 0.001 Ry/*a*_*B*_, where *a*_*B*_ is the Bohr radius.

## Additional Information

**How to cite this article**: Jue, M. *et al*. The determining factor of a preferred orientation of GaN domains grown on *m*-plane sapphire substrates. *Sci. Rep*. **5**, 16236; doi: 10.1038/srep16236 (2015).

## Figures and Tables

**Table 1:** Preferred orientations of GaN grown in one of the divisions.

Preferred Orientation	(1 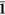 00)	(1 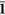 0 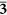 )	(1 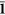 00) & (1 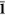 0 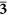 )	N.A.
Division (FROA)				
IV (0.403)	98.2 (%)	0.0 (%)	1.2 (%)	0.6 (%)
I (0.227)	64.5 (%)	7.7 (%)	26.8 (%)	1.0 (%)
V (0.227)	65.8 (%)	8.7 (%)	18.9 (%)	6.6 (%)
II (0.128)	0.0 (%)	100.0 (%)	0.0 (%)	0.0 (%)
VI (0.101)	0.0 (%)	100.0 (%)	0.0 (%)	0.0 (%)
III (0.0567)	0.0 (%)	100.0 (%)	0.0 (%)	0.0 (%)

Note that the percentage value refers to the percentage of number of openings with the specified preferred orientation. The domains coded as blue and cyan in Fig. 3 are both counted as the (1
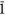
00)-oriented domains, so that only four categories of preferred orientations are listed in this table although the domains were classified into five groups in Fig. 3.
